# The Surgeon-General’s Annual Report

**Published:** 1882-12

**Authors:** 


### THE SURGEON GENERAL'S ANNUAL REPORT.

The annual report of Surgeon General Crane, for the current year, contains a carefully tabulated statement of receipts and expenditures in his department; statiscal data concerning the health and physical condition of the army during the past year; a general review of the work in the record and pension divisions, and other matters of interest pertaining to his office.

The library of the Surgeon General's office, he says, is devoted entirely to medicine and its branches. The works contained therein are not duplicated in any other library in Washington, and no advantage, he claims, would accrue from merging this library into any other, for it requires the special services of a skilled medical officer to make it useful and to retain for it the interest of the medical profession of the country, to which its completeness is largely due.

The additions to the library for the past year numbered over three thousand volumes and thirty-five hundred pamphlets; making a total of fifty-seven thousand volumes and over sixty-three thousand pamphlets.

The use of the library by the medical profession is steadily increasing. Over three hundred requests for information were received during the year from all parts of the United States and one thousand letters were required to answer these correspondents.

The manuscript of volume four of the Index Catalogue is nearly ready and the first part is passing through the press. The third surgical volume of the Medical and Surgical History of the war, will be finished before the adjournment of the approaching session of congress.

The report urges upon congress the necessity for a new and fire-proof building suitable for the proper accommodation of the Army Medical Museum and Library. The present quarters are unfit for the purposes intended and are insecure against loss by accident or fire, and the destruction of the contents of the building would entail an irreparable loss, not only upon America but upon the world.

Out of two hundred and sixteen candidates who appeared before the Army Examining Board, for positions as assistant surgeons, thirty-nine were found qualified, fifty-one were rejected and one hundred and twenty-six withdrew after partial examination.

The late Surgeon General and three surgeons have been retired, and three surgeons and three assistant surgeons have died during the year. Thirteen assistant surgeons have been appointed. A vacancy now exists in the office of Assistant Surgeon General, and six vacancies exist in the grade of assistant surgeon.

